# Altered Food-Cue Processing in Chronically Ill and Recovered Women with Anorexia Nervosa

**DOI:** 10.3389/fnbeh.2015.00046

**Published:** 2015-02-27

**Authors:** Nicole Sanders, Paul A. M. Smeets, Annemarie A. van Elburg, Unna N. Danner, Floor van Meer, Hans W. Hoek, Roger A. H. Adan

**Affiliations:** ^1^Altrecht Eating Disorders Rintveld, Altrecht Mental Health Institute, Utrecht, Netherlands; ^2^Brain Center Rudolf Magnus, University Medical Center Utrecht, Utrecht, Netherlands; ^3^Image Sciences Institute, University Medical Center Utrecht, Utrecht, Netherlands; ^4^Division of Human Nutrition, Wageningen University and Research Center, Wageningen, Netherlands; ^5^Faculty of Social Sciences, University of Utrecht, Utrecht, Netherlands; ^6^Parnassia Psychiatric Institute, The Hague, Netherlands; ^7^Department of Psychiatry, University Medical Center Groningen, Groningen, Netherlands; ^8^Department of Epidemiology, Mailman School of Public Health, Columbia University, New York, NY, USA

**Keywords:** anorexia nervosa, recovery, functional magnetic resonance imaging, food viewing

## Abstract

Anorexia nervosa (AN) is a severe mental disorder characterized by food restriction and weight loss. This study aimed to test the model posed by Brooks et al. (2012a,b) that women suffering from chronic AN show decreased food-cue processing activity in brain regions associated with energy balance and food reward (bottom-up; BU) and increased activity in brain regions associated with cognitive control (top-down; TD) when compared with long-term recovered AN (REC) and healthy controls (HC). Three groups of women, 15 AN (mean illness duration 7.8 ± 4.1 years), 14 REC (mean duration of recovery 4.7 ± 2.7 years) and 15 HC viewed alternating blocks of food and non-food images preceded by a short instruction during functional magnetic resonance imaging (fMRI), after fasting overnight. Functional region of interests (fROIs) were defined in BU (e.g., striatum, hippocampus, amygdala, hypothalamus, and cerebellum), TD (e.g., medial and lateral prefrontal cortex, and anterior cingulate), the insula, and visual processing areas (VPA). Food-cue processing activation was extracted from all fROIs and compared between the groups. In addition, functional connectivity between the fROIs was examined by modular partitioning of the correlation matrix of all fROIs. We could not confirm the hypothesis that BU areas are activated to a lesser extent in AN upon visual processing of food images. Among the BU areas the caudate showed higher activation in both patient groups compared to HC. In accordance with Brooks et al.’s model, we did find evidence for increased TD control in AN and REC. The functional connectivity analysis yielded two clusters in HC and REC, but three clusters in AN. In HC, fROIs across BU, TD, and VPA areas clustered; in AN, one cluster span across BU, TD, and insula; one across BU, TD, and VPA areas; and one was confined to the VPA network. In REC, BU, TD, and VPA or VPA and insula clustered. In conclusion, despite weight recovery, neural processing of food cues is also altered in recovered AN patients.

## Introduction

Anorexia nervosa (AN) is a mental disorder with a lifetime prevalence rate of 1–2% in women (Hudson et al., 2007; Smink et al., 2013) and poor outcome. Of all mental disorders, AN is among those with the highest mortality rates (Arcelus et al., 2011; Brooks et al., 2012a).

Food restriction and weight loss in AN are most striking (Santel et al., 2006). However, these characteristics remain poorly understood. It has been suggested that the motivation to eat may be overruled by higher order brain centers preoccupied with fears of eating (Brooks et al., 2012a; Frank, 2013). Despite being in negative energy balance, AN patients remain highly restraint and even show aversion and disgust to food images, which is driven by their fear of weight gain (Uher et al., 2004).

In many mental disorders, researchers have proposed bottom-up (BU) and top-down (TD) models to explain information processing (Cohen et al., 2012; Hirsch and Mathews, 2012). BU processing involves the flow of information from periphery to the cortex. The TD processing involves the selection of inputs most likely to be relevant based on an individual’s experience and expectations (Epstein et al., 2001).

When processing information about food, several different neural circuits, involving motivational drive, satiety, and anticipation to food, become active (Kelley et al., 2005). Peripheral hormones, such as leptin and ghrelin, signal information on current metabolic state to structures, such as the hypothalamus and the mesolimbic dopamine system. The latter consists of the ventral tegmental area that projects to cortico-limbic structures, such as the nucleus accumbens, hippocampus, and amygdala. This widespread signaling modulates various reward signals, important for feeding behavior (van Zessen et al., 2012).

The TD processing and cognitive evaluation and control of eating behavior are influenced by a variety of factors that include learned appraisal of food stimuli (Berridge, 2009) and underlying trait alterations (Kaye et al., 2013). In AN patients, this appraisal of food stimuli has been suggested to induce fear, as food promotes weight gain (Kaye et al., 2009).

**Figure 1:**
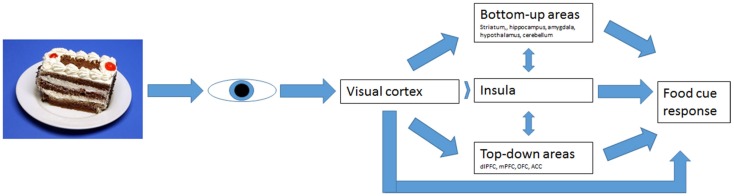
**Bottom-up and top-down regulation of visual food-cue responses according to Brooks’ model**.

In AN, the BU information processing was hypothesized to be decreased and TD processing increased, suggesting a diminished drive to eat and increased cognitive control over food cues, respectively (Brooks et al., 2012a; Kaye et al., 2013). It is hard to find consensus on brain areas involved in TD and BU information processing. Brooks et al. (2012a) have tried to define these systems (Figure 1) and tested its validity for eating disorders. Since it offers a clear hypothesis, we aimed to test this model here.

Brooks et al. (2011) found reduced activation in AN in the bilateral cerebellar vermis, following food images, as well as an increase in the dorsolateral prefrontal cortex (dlPFC). This increase in the dlPFC was also found by Uher et al. (2003, 2004.) These findings suggest decreased activity in BU processing and an increase in TD processing. Thus, AN patients may receive less strong appetitive signaling and react with more fear and aversion to food (Brooks et al., 2012a).

There is, however, limited consensus between studies, most likely due to the difference in experimental design. We used the same paradigm as used previously by Uher et al. (2003, 2004). Since Santel et al. (2006) found the largest differences between healthy controls (HC) and patients during a hungry state, we decided to test our participants after an overnight fast.

Anorexia nervosa patients are known to show an attentional bias (Blechert et al., 2011) as well as avoidance to food cues (Giel et al., 2011). Since our participants were asked to actively view food cues, we included visual attention areas as described by Fan et al. (2002) to test for attentional differences.

Little is known about brain functioning in long-term recovered AN patients. Therefore, we aimed to study a group of women recovered from AN to study information processing of food after being fully weight and menstrual cycle recovered. As most patients relapse in the first 18 months after weight and menstrual cycle recovery (Carter et al., 2004), we included women that were long-term recovered.

A relatively new field of interest is the study of functional connectivity. One type of functional connectivity analysis studies the temporal correlation between different brain regions during task performance. Areas displaying a high correlation in activation are suggested to be functionally connected regions (Zhang and Raichle, 2010; Favaro et al., 2012). This type of analysis may help establish functional networks, which provides additional information on differences in food-cue processing in our study populations. It allows one to test in how far the brain areas activated during neural processing of food cues are functionally connected in the same way in HC, AN, and REC. Alterations in functional connectivity during task performance have been reported in various neurological and mental disorders, including depression and anxiety (Avery et al., 2013; Clauss et al., 2014).

In AN, functional connectivity has been studied predominantly at rest (Zhang and Raichle, 2010; Amianto et al., 2013; Cowdrey et al., 2014). Less attention has been directed to the examination of neural networks during active task performance, which is important because certain connections during a task may not be evident at rest (Pezawas et al., 2005; Bilek et al., 2013).

In this study, our primary aim was to test activation of the BU and TD systems in currently ill as well as recovered women with AN in comparison with HC women. In concordance with literature, we hypothesized that in response to viewing food images, we would find less activation in BU areas and increased activation in TD areas during cognitive processing of food images in the AN group. As women recovered from AN have normalized their weight and eating behavior, we expected that they would also have normalized brain responses to food image evaluation. However, if the recovered group differs in their response to controls, we would expect them to show increased activation of the TD areas, suggesting food evaluation to be more cognitively driven than in HC.

Secondarily, we aimed to test functional connectivity between all *a priori* defined areas during cognitive processing of food images and images of objects. We hypothesized that as BU and TD processing are aberrant in AN patients, functional connectivity will be disturbed in AN patients as well.

## Materials and Methods

### Subjects

Three groups of women participated in this study; 15 healthy weight women without any history of psychiatric disorders (HC: mean age 25.8 ± 5 years); 15 women with chronic AN (mean age 25.6 ± 5 years; mean illness duration: 7.8 ± 4.1 years), and 15 women recovered from AN (REC: mean age 24.3 ± 5 years; mean duration of recovery: 4.7 ± 2.7 years), diagnosed by a psychiatrist according to DSM-IV criteria. The AN group consisted of in- or outpatients in a specialized eating disorder clinic (Altrecht Eating Disorders Rintveld). The REC group consisted of a group of former in- and outpatients screened by a resident in psychiatry. See Table 1 for demographics.

**Table 1:** **Subject characteristics**.

Measure^a^	Group		
	HC	AN	REC
*n*	15	15	14
Age (years)	25.8 (5)	25.6 (5)	24.3 (5)
Diagnosis AN-R/AN-P	NA	9/6	10/4
Illness duration (years)	NA	7.8 (4.1)^c^	4.1 (2.7)
Duration of recovery (years)	NA	NA	4.7 (2.7)
BMI (kg/m^2^)	21.5 (2.3)	14.5 (1.7)^b^,^c^	21.1 (1.9)
Body fat percentage	25 (5)	11 (5)^b^,^c^	25 (5)
Trait anxiety (STAI score)	31 (6)	48 (3)^b^	48 (4)^b^
State anxiety (STAI score)	31 (5)	47 (4)^b^	47 (4)^b^
BDI total score	1 (3)	39 (6)^b^	9 (10)^d^
EDE-Q restrained eating	0.39 (0.66)	3.93 (0.85)^b^,^c^	0.98 (0.75)
EDE-Q eating concern	0.17 (0.26)	3.91 (0.61)^b^,^c^	1.04 (0.93)^b^
EDE-Q weight concern	0.28 (0.41)	4.7 (1.36)^b^,^c^	2.32 (1.5)^b^
EDE-Q shape concern	0.33 (0.35)	5.44 (0.78)^b^	2.52 (1.30)^b^,^c^
EDE-Q global score	0.12 (0.2)	2.63 (3.5)^b^,^e^	0.63 (0.6)
VAS anxiety start (0:100)	16.7 (15)	32.5 (25)	25.3 (24)
VAS anxiety end (0:100)	2.7 (4.6)	26.9 (6.7)^d^	16.7 (19)
VAS desire to eat start (0:100)	76 (8.3)	38.8 (32)^b^	46.0 (26.7)^b^
VAS desire to eat end (0:100)	85.3 (11)	26.9 (24)^b^	46.0 (32)^b^

*^a^Values shown are mean (SD)*.

*^b^Significant differences in *post hoc* tests corrected for multiple comparisons (*P* < 0.05): significantly different from HC *P* < 0.01*.

*^c^Significant differences in *post hoc* tests corrected for multiple comparisons (*P* < 0.05): significantly different from REC *P* < 0.01*.

*^d^Significant differences in *post hoc* tests corrected for multiple comparisons (*P* < 0.05): significantly different from HC *P* < 0.05*.

*^e^Significant differences in *post hoc* tests corrected for multiple comparisons (*P* < 0.05): significantly different from REC *P* < 0.05*.

*HC, healthy control; AN, chronically ill anorexia nervosa patients; REC, women recovered from AN; AN-R, restrictive subtype; AN-P, purging subtype*.

Exclusion criteria for all groups were smoking, left-handedness, major medical illness, or current use of dopaminergic or serotonergic medication. Furthermore, exclusion criteria were a history of neurologic disorders, current pregnancy, claustrophobia, and metal objects in the body that would interfere with the fMRI.

Additional exclusion criteria for REC and HC were BMI (in kg/m^2^) <18.5 or >25 at time of screening; current dieting or weight loss; and a behavior pattern of restrained eating (according to EDE-Q).

The Utrecht Medical Center Medical Ethical Committee approved the study. Participants provided written informed consent.

### Study procedures

Participants, if menstruating, were asked to participate in the first 2 weeks of their menstrual cycle, since leptin is lowest during this period, presumably resulting in lower food reinforcement (McNeil and Doucet, 2012). Some participants used an intra-uterine device, preventing them from having their periods. Furthermore, all chronic AN patients had – by definition – amenorrhea or used oral anticonceptives. Participants began fasting at 10 p.m. on the night prior to the study visit, since Santel et al. (2006) showed that, while rating the salience of food, hungry AN patients displayed active suppression of attention to food cues, facilitating their abilities to fast. This active suppression was not found in satiated patients. All subjects arrived at the University Medical Center Utrecht between 7 and 8.30 a.m. for the fMRI scan after which body measures were obtained.

### Measures

#### Body weight and eating behavior

Weight and body composition were measured using a bio-impedance scale. BMI was calculated after measuring height and current weight. To identify aberrant eating behavior the EDE-Q (Fairburn and Beglin, 1994) was administered. This is a 29-item scale designed to identify disturbed eating patterns as well as weight and shape concerns.

#### Stimulus material: food images

The fMRI paradigm, including the images, used in this study has been used before in another study with eating disorder patients at the Institute of Psychiatry in London (Uher et al., 2003, 2004); King’s College London (Institute of Psychiatry), London, United Kingdom. A pilot was performed to test whether the food images were appropriate for the Dutch population. The food images consisted of 60 color photographs of high and low calorie, sweet and savory food, presented on white plates on a blue background. The food images were presented in random order. The non-food (NF) images consisted of 60 color photographs of objects on a white plate with a blue background. Images were selected and matched according to color and visual structure.

#### Imaging paradigm

Participants were scanned on a 3T Philips Achieva using a 3D-PRESTO SENSE sequence (TR/TE = 22.5/33 ms, flip = 10°, voxel size = 4 mm × 4 mm × 4 mm, dynamic scan duration = 608 ms). During a 12 min functional scan participants were presented with images in six alternating 30-s blocks. Each block consisted of 10 food (F) or NF images preceded by a short instruction, e.g., “Imagine eating/using the food/object presented,” which was shown on the screen before each block.

#### Statistical analysis

Functional magnetic resonance imaging data were preprocessed and analyzed with the SPM8 software package [Wellcome Department of Imaging Neuroscience^1^, London, United Kingdom] in conjunction with the MarsBar toolbox^2^ run with MATLAB7.9 (The Mathworks Inc., Natick, MA). The functional volumes of every subject were realigned to the first volume of the first run, globally normalized to Montreal Neurological Institute space (MNI space) retaining 4 mm × 4 mm × 4 mm voxels, and spatially smoothed with a Gaussian kernel of 8 mm full width at half maximum. A statistical parametric map was generated for every subject by fitting a box car function to each time series, convolved with the canonical hemodynamic response function. Data were high-pass filtered with a cut-off of 128 s. Four conditions were modeled: viewing foods, viewing NFs, rating desire to eat and anxiety on a visual analog scale (VAS), and reading instructions before every block of foods and NFs. For every subject, parameters were estimated for the comparison (contrast) food minus NF viewing (F > NF).

To test our hypothesis, a whole-brain statistical F-map was created by performing an ANOVA with image type (F and NF) and time as independent variables per group (HC, AN, and REC). We used a region of interest (ROI) approach as, e.g., employed by Mehta et al. (2012) and Griffioen-Roose et al. (2013), which combined *a priori* anatomical areas of interest with a functional criterion based on a minimum level of responsiveness to food cues. *A priori* anatomical ROIs (see Appendix 1 in Supplementary Material) were chosen based on Frank (2013) and Kaye et al. (2013) and integrated in the model of Brooks et al. (2012a). For BU areas, mesolimbic areas as the striatum, hippocampus, amygdala, hypothalamus, and cerebellum have been defined. The TD areas have been defined by the dlPFC, medial prefrontal cortex (mPFC), orbitofrontal cortex (OFC), and the anterior cingulate cortex (ACC). The insula was also taken into account as the bridge between TD and BU (Brooks et al., 2012a). To test for the effects of attention, we included attentional areas as defined by Fan et al. (2002) (e.g., visual cortex, superior frontal cortex, inferior parietal cortex, superior parietal cortex, and the posterior cingulate cortex). For all structures, anatomical masks were constructed using the Wake Forest University Pickatlas toolbox (Maldjian et al., 2003). With exception of the prefrontal cortex and cerebellum, all mask images were dilated 2 voxels to account for anatomical variation and smoothing effects.

To identify functional ROIs (fROIs), both created maps were thresholded at a significance level of *P* < 0.05 and a cluster size *k* > 9 contiguous voxels. The identified fROIs are shown in Appendix 1 in Supplementary Material.

Subsequently, for all subjects the mean beta value in each fROI was calculated with the use of MarsBar (see footnote text 2), and entered into an one-way analyses of variance (ANOVA) in statistical package for the social sciences (SPSS) version 20, using Tukey, HSD correction was used to correct for multiple comparisons in the *post hoc* t-test. To unveil which groups showed a significant response (F > NF), a one-sample t-test (sign different from 0) was performed for each group. This fROI approach represents an unbiased way to test *a priori* hypotheses and avoids problems of circularity (Mehta et al., 2012; Griffioen-Roose et al., 2013).

#### Functional connectivity analysis

For each group, Pearson correlation coefficients were computed between the mean beta values in each fROI as a measure of functional connectivity. This resulted in a <24 fROIS × 24 fROIs > correlation matrix for each group (Appendix 3A–C in Supplementary Material). Next, the level of community organization of each of these matrices was computed by means of a Louvain modularity algorithm (Rubinov and Sporns, 2009), unfolding a complete hierarchy of connected functional clusters, resulting in a modular partitioning of each of the correlation matrices.

## Results

The fROI results are described below per system. Results for all fROIs are shown in Table 2.

**Table 2:** **Between group differences in food-cue activation**.

fROI	Mean beta value (±SEM)			*F* value
	HC	AN	REC	
**BOTTOM-UP**				
R caudate nucleus	0.036 (0.18)	0.092 (0.22)	0.220 (0.21)^a^	3.076
L hippocampus	0.038 (0,18)	0.125 (0.16)	0.164 (0.18)	2.050
R hippocampus	0.117 (0.20)	0.160 (0.22)	0.203 (0.20)	0.623
Hypothalamus	0.134 (0.21)	0.126 (0.18)	0.026 (0.25)	1.162
Vermis	0.146 (0.31)	0.101 (0.35)	0.306 (0.18)	1.925
Vermis 2	0.034 (0.17)	0.170 (0.22)	0.209 (0.21)	3.039
L cerebellum	0.136 (0.17)	0.248 (0.26)	0.214 (0.25)	0.963
R cerebellum	0.057 (0.18)	0.366 (0.33)^a^	0.324 (0.33)	4.259
R cerebellum 2	−0.035 (0.27)	0.144 (0.20)	0.191 (0.17)^a^	4.520
**INSULA**				
R insula	0.140 (0.23)	0.107 (0.26)	0.185 (0.16)	0.452
**TOP-DOWN**				
L middle frontal gyrus	−0.142 (0.12)	−0.018 (0.12)^a^	−0.001 (0.11)^b^	5.997
R middle frontal gyrus	0.098 (0.21)	0.157 (0.14)	0.206 (0.05)	1.311
R middle frontal gyrus 2	0.062 (0.21)	0.056 (0.18)	0.177 (0.21)	1.628
**VISUAL PROCESSING**				
R cuneus	−0.126 (0.18)	−0.101 (0.23)	−0.134 (0.19)	0.111
L precuneus	−0.109 (0.18)	−0.111 (0.16)	−0.104 (0.20)	0.006
R precuneus	0.148 (0.27)	−0.126 (0.20)^a^	−0.079 (0.30)	4.776
R superior frontal gyrus	0.067 (0.20)	−0.102 (0.13)^a^	−0.042 (0.11)	4.857
R lingual gyrus	0.188 (0.27)	0.226 (0.13)	0.302 (0.17)	1.188
L inferior parietal cortex	−0.046 (0.19)	0.077 (0.13)	0.095 (0.20)	2.800
R inferior parietal cortex	0.203 (0.28)	0.177 (0.28)	0.163 (0.18)	0.095
L post central gyrus	−0.111 (0.79)	−0.037 (0.09)	0.104 (0.24)^b^	5.274
L superior temporal gyrus	−0.080 (0.17)	−0.104 (0.14)	−0.076 (0.15)	0.143
R superior temporal gyrus	−0.100 (0.15)	−0.136 (0.16)	−0.124 (0.17)	0.197
R posterior cingulate cortex	−0.046 (0.19)	−0.199 (0.24)	−0.118 (0.17)	2.145

*HC, healthy control; AN, chronically ill anorexia nervosa patients; REC, women recovered from anorexia nervosa; L, left; R, right*.

*^a^Significantly different from HC *P* < 0.05*.

*^b^Significantly different from HC *P* < 0.01*.

### Bottom-up system

**Figure 2:**
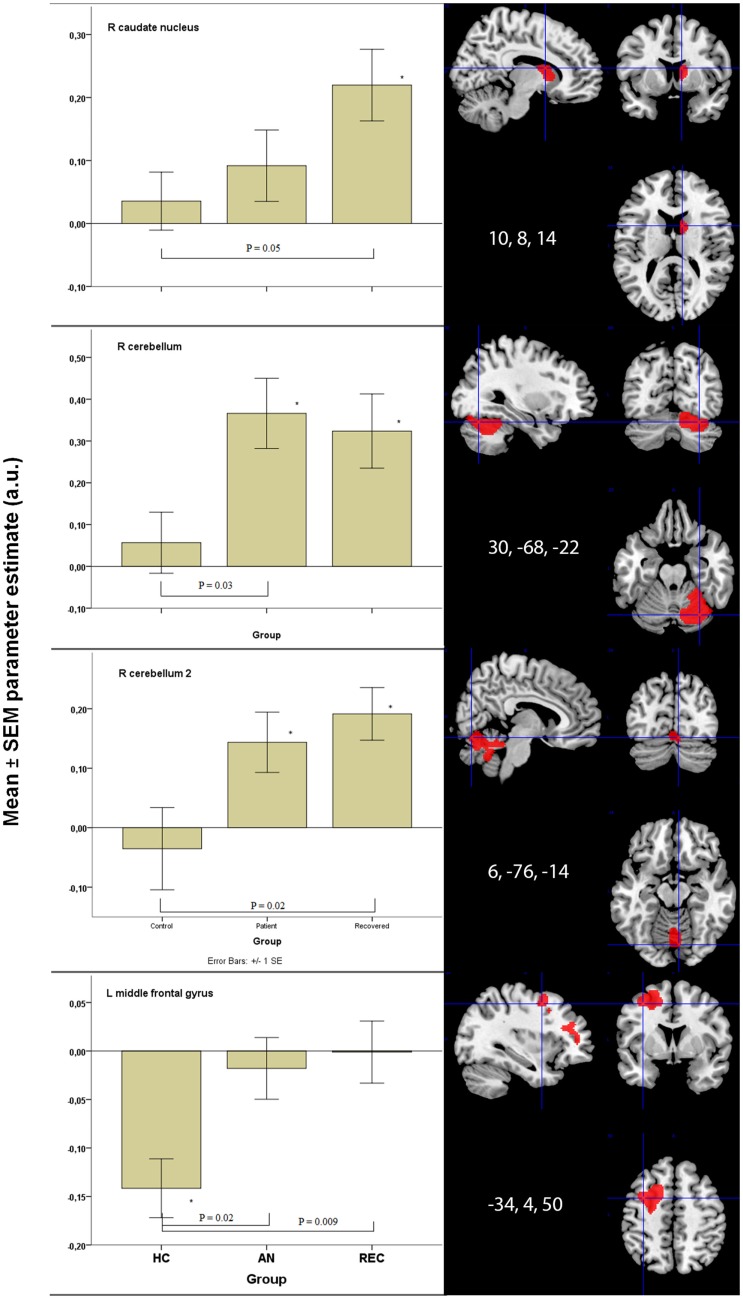
**Significant between group differences in food-cue activation results in bottom-up and top-down areas**. *p < 0.05 when comparing food to non-food images. HC, healthy control; AN, anorexia nervosa; REC, recovered group; SEM, standard error of the mean; a.u., arbitrary units.

There was a significant difference in activation of the right caudate nucleus between the HC and REC group. HC showed no activation, whereas the REC showed significant activation. Activation in the AN group did not differ from either group (Figure 2).

There were two fROIs with a similar pattern in the right cerebellum (Figure 2). In the first area, there was a significant difference between the HC group and the AN group and in the second area the REC group showed a significant difference with the HC group. In both these areas, activation was significant in both AN groups, but not in the HC group.

The left hippocampus and vermis displayed the highest activation in the AN groups. In these areas, there was no activation in the HC, whereas the AN and REC groups showed significant activation.

In the hypothalamus, there was significant activation during food image viewing in the HC and AN group. This activation was not found in the REC group. Even though the groups differed in food-cue activation, there was no significant difference between the groups.

There was no difference between the three groups in both right hippocampus and left cerebellum.

### Insula

We found no group difference. The Insula showed significant activation in both REC group and HC group, but no activation in the AN group.

### Top-down system

The left middle frontal gyrus displayed significant activation in the HC group, but not in both AN groups. This deactivation in HC group was significantly different from the AN group and the REC group (Figure 2).

In the right middle frontal gyrus, there was an opposite effect, where both AN groups showed activation. In the HC group, this activation was absent. Despite the difference in food-cue activation within the three groups, there was no significant difference between the three groups.

### Visual processing areas

**Figure 3:**
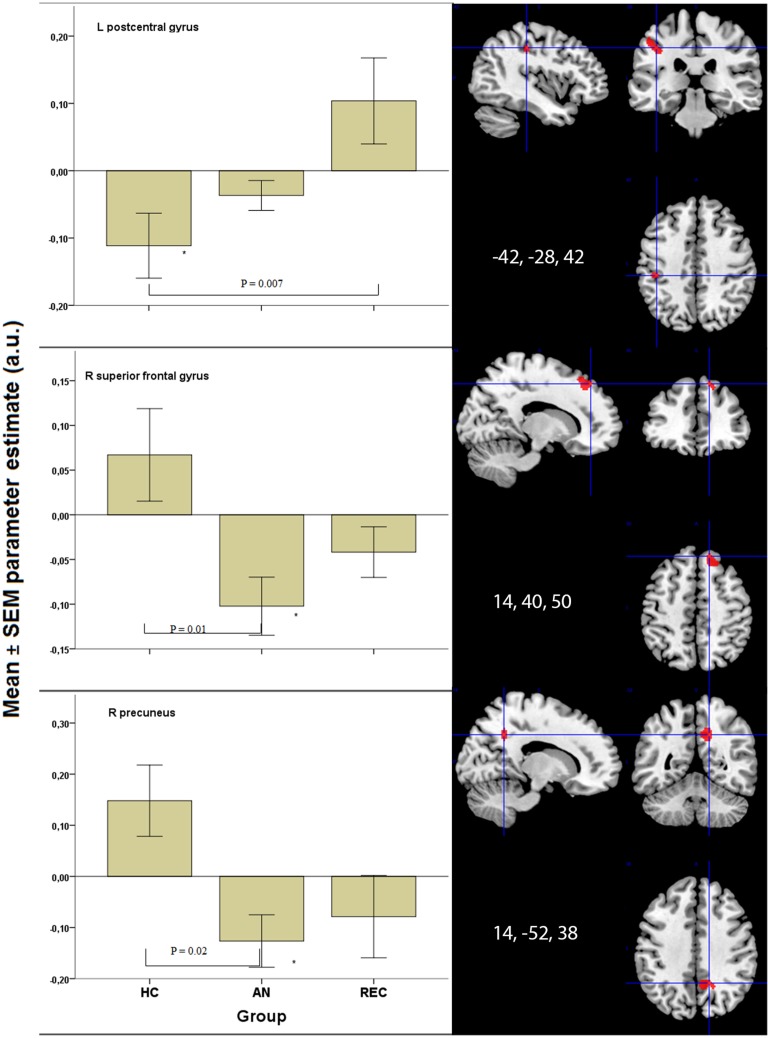
**Significant results in visual processing areas**. *p < 0.05 when comparing food to non-food images. HC, healthy control; AN, Anorexia Nervosa; REC, recovered group; SEM, standard error of the mean; a.u., arbitrary units.

In the superior frontal gyrus, there was deactivation in the AN group, and no activation in the HC and REC group. There was a significant difference between the AN and HC group (Figure 3).

The right precuneus displayed deactivation in the AN groups only, with a significant decrease in activation in AN when compared with HC (Figure 3). In the left post central gyrus, there was deactivation in the HC group, but not in the AN groups. There was a significant difference between the HC and the REC group (Figure 3).

In the left inferior parietal cortex, there was activation in the AN group only, with no significant difference between the three groups. In the right posterior cingulated cortex, there was deactivation in both AN groups, but not in the HC group, although there were no significant differences between the groups.

There was a similar pattern in the right lingual gyrus, right inferior parietal cortex, and right superior temporal cortex. In the right cuneus, as well as the left precuneus, there was deactivation in AN and HC, but not in REC, although there was no difference between the three groups. The left superior temporal cortex deactivated to food images only in the AN group. No significant differences were found between the three groups.

### Functional connectivity

For each group, the fROIs, i.e., the food-responsive clusters, were partitioned into modules based on the strength of the correlations between the mean beta values in each fROI.

Partitioning of the fROI correlation matrices of the control and recovered groups resulted in two modules, while in the patient group there were three modules. These modules are tabulated in Table 3 and depicted in Appendix 2 in Supplementary Material. In HC, there was no predominance of any category in either of the two identified modules. In AN, the third module (A3) was composed solely of visual processing areas (VPA). In REC, all BU and TD areas were clustered in one module (R1), while the insula and most VPA were in the other module (R2).

**Table 3:** **Functional clustering of fROIs for each group**.

fROI	HC	AN	REC	Classification
R caudate nucleus	H 1	A 1	R 1	BU
Hypothalamus	H 1	A 1	R 1	BU
R middle frontal gyrus	H 1	A 2	R 1	TD
R middle frontal gyrus	H 1	A 2	R 1	TD
R inferior parietal cortex	H 1	A 2	R 2	VPA
L precuneus	H 1	A 3	R 2	VPA
R precuneus	H 1	A 3	R 2	VPA
L superior temporal cortex	H 1	A 3	R 2	VPA
R superior temporal gyrus	H 1	A 3	R 2	VPA
L hippocampus	H 2	A 1	R 1	BU
R hippocampus	H 2	A 1	R 1	BU
Vermis	H 2	A 1	R 1	BU
R cerebellum	H 2	A 1	R 1	BU
Vermis	H 2	A 2	R 1	BU
L cerebellum	H 2	A 2	R 1	BU
R cerebellum	H 2	A 2	R 1	BU
R insula	H 2	A 1	R 2	INS
L middle frontal gyrus	H 2	A 1	R 1	TD
R superior frontal cortex	H 2	A 3	R 2	VPA
R lingual gyrus	H 2	A 2	R 1	VPA
L inferior parietal cortex	H 2	A 2	R 1	VPA
L postcentral gyrus	H 2	A 2	R 1	VPA
R cuneus	H 2	A 3	R 2	VPA
R posterior cingulate cortex	H 2	A 3	R 2	VPA

*The numbers 1, 2 and 3 denotes number of the cluster*.

*HC, healthy control; AN, chronically ill anorexia nervosa patients; REC, women recovered from Anorexia Nervosa; R, right; L, left; BU, bottom-up area; TD, top-down area; INS, insula; VPA, visual processing/attention*.

## Discussion

In this study, we tested whether food-cue processing in brain regions associated with BU (from periphery to cortex) is downregulated and TD processing is upregulated in currently ill and recovered AN patients.

In contrast to the proposed model (Brooks et al., 2012a), we found increased activity in the caudate nucleus (BU area) especially in the recovered AN group (REC), when comparing with HC. We could confirm an increase in activation in the cerebellum, but overall our findings do not support decreased activity in the BU system as Brooks et al. proposed. For the TD areas, we found evidence for increased activity, in line with Brooks et al. (2012a).

Our main findings in AN were increased activation in the BU system, but only in the right cerebellum. We found increased activation in the TD system, but only in the middle frontal gyrus and decreased activation in the VPA in AN.

In REC, we found increased activation in the caudate nucleus and right cerebellum in the BU system. In the TD system, we found increased activation in the left middle frontal gyrus (similar to AN). In the VPA, we found a strong increase in activation in the left post central gyrus.

### Bottom-up system

Zhu et al. (2012) described increased activation in the right caudate nucleus in their meta-analysis of fMRI studies in AN. We, however, found activation in the REC group only. As Zhu et al. (2012) suggest, the right caudate is involved in emotions of disgust. The HC report desire to eat (Table 1) and show no activation in the caudate nucleus. Activation in the right caudate nucleus in REC women might suggest disgust, as Zhu et al. (2012) suggest, and can be related to continuous worry about shape and weight (as measured by the EDE-Q subcategories weight and shape concern; see Table 1) even though food intake and body weight have been restored, and REC report desire to eat (Table 1).

The cerebellum and vermis are thought to have a prominent role in feeding behavior (Zhu and Wang, 2008). We found that the anorexia groups show increased activation in these areas after an overnight fast. This suggests an increased appetite response when asked to imagine eating foods. This finding is in line with Brooks et al. (2012b), who found decreased food viewing activation in AN patients who just ate, indicating increased reactivity to food when AN patients are fasting. Amianto et al. (2013) described that the cerebellum is also involved in emotion regulation in AN patients. This suggests that instead of only enhancing appetite, food-cue processing might be a more emotional process in AN patients.

Interestingly, we found activation in the hypothalamus in both the HC and AN groups, but not in REC. This activation in HC appears to reflect the increased appetite healthy women perceive after an overnight fast. The AN group is extremely emaciated (as confirmed by their low leptin levels), possibly explaining why they also show activation to food images in the hypothalamus. Given the history of extremely low BMI in recovered women, we cannot exclude that at the level of the hypothalamus, a normal weight is perceived as positive energy balance and that this may contribute to the lack of response to food images after an overnight fast in hypothalamus. Thus a shift in set point may explain why an overnight fast may not be perceived as being in a negative energy balance, explaining why REC do not show any difference in activation in the hypothalamus between food and NF images.

We demonstrated activation of the left hippocampus in both AN groups, but not in HC. The hippocampus is involved in contextual memory and associated with anxiety and depression (Nunn et al., 2008; Mineur et al., 2013). We found similar activation in the right hippocampus in all groups, possibly indicating that all groups were engaged in visual processing and activating visual memory in a similar manner. The left hippocampus, however, is involved in sequential processing (Nunn et al., 2008), resembling higher activation in working memory during food image processing. Skipping breakfast led to increased hippocampal activation in response to food images in overweight to obese adolescent girls (Leidy et al., 2011). Eating would lead to a reduction in activation, suggesting its involvement in perceived hunger and food motivation (Leidy et al., 2011). Curiously, despite lower reported desire to eat (Table 1), the hippocampal activation is higher in both AN groups compared to HC, suggesting active food information processing despite lack of reported desire to eat.

### Top-down

Within the TD areas, we found no activation in the left middle frontal gyrus in both AN and REC groups but decreased activation in HC. The right middle frontal gyrus was activated in REC and AN, but not in HC. Rosenbaum et al. (2008) found increased activation in both left and right middle frontal gyrus during food image processing after a 10% weight loss in six obese subjects.

Furthermore, Wierenga et al. (2014) found a comparable group difference in this same part of the middle frontal gyrus. Individuals with remitted AN showed increased activation to monetary decision-making compared to normal weight controls in both the hungry and the satiated state. Both studies suggest that increased activation reflects enhanced cognitive control (Rosenbaum et al., 2008; Wierenga et al., 2014). Rosenbaum further suggests that this increased activation might imply increased emotional responses to food (Wierenga et al., 2014). Because we found activation of the same part of the middle frontal gyrus during food image processing in AN and REC, this may reflect an increased emotional response to foods, which may be related to the inhibition of food intake.

### Visual processing areas

We found decreased activation of the right superior frontal gyrus in AN in response to visual food cues. This decreased activation is in line with the deactivation. Rothemund et al. (2011) found studying food image processing. In this study, women with AN and normal weight controls were shown images of high versus low-calorie foods, eating utensils (e.g., plates, knives, forks), and neutral control images. They correlate activation in the right superior frontal gyrus to “obsessive thoughts” measured by the Y-BOCS and suggested that AN patients display strong response inhibition and, therefore, less goal directed behavior toward food (Rothemund et al., 2011). Our coordinates resemble one of their coordinates in the superior frontal gyrus, suggesting the same holds true for our population.

In the REC group, the increased activation in the right lingual gyrus is in line with the findings from Brooks et al. (2011, 2012b), who also found a significant increase in activation in this area during food image viewing in AN patients compared to controls. Rosenbaum et al. (2008) found increased activation after weight loss in obese subjects, suggesting increased attention to food cues.

The precuneus is thought to be involved in self-relevant mentalizing and interoception (McFadden et al., 2014). In this study, we found a decreased precuneus response in recovered and chronic AN patients when viewing food images. This is in line with the decreased precuneus activation, which has been implicated in suppression of cravings for food (Zhu et al., 2012; Yokum and Stice, 2013) and further supported by Cavanna and Trimble (2006) who state that the precuneus is involved in internally guided attention.

We found increased activation in REC in the left post central gyrus, when compared with HC. The left post central gyrus is thought to be involved in systematic integration of somatic and visual information (Iwamura, 1998). Obese children and adolescents displayed greater activation viewing food logos and fast food commercials, respectively, when compared with normal weight children and adolescents (Bruce et al., 2012; Gearhardt et al., 2013). Interestingly, REC show a similar increase in activation of the left post central gyrus to food viewing, even though they do not display an increase in desire to eat (Table 1). This suggests that individuals suffering from eating disorders are not fully able to integrate visual information with somatic information, possibly leading to either inhibition or disinhibition of food intake. Markedly, this activation was not seen in AN.

### Cluster analysis

The functional connectivity analysis showed distinctly different clustering of the fROIs in all three groups. In the REC group, all BU and TD fROIs were clustered in one module and the insula and most VPA in another.

Notably, the AN group had three rather than two modules. Noteworthy is that all areas in the “extra” module in AN are VPA and in all but one of these areas AN show the strongest response (i.e., either strongest activation or strongest decrease in activation) to food cues. Within this third module, the precuneus and superior frontal gyrus were both suggested to be involved in suppression of food craving (Zhu et al., 2012; Yokum and Stice, 2013) and response inhibition (Rothemund et al., 2011). Potentially, this altered functional connectivity is associated with the inhibition of food intake.

Moreover, a resting state fMRI study found that both ill and recovered anorexia patients had decreased functional connectivity in the ventral visual network (Favaro et al., 2012). A recent study also found increased resting state connectivity in cognitive control areas (dlPFC/IFG) in recovered AN patients (Cowdrey et al., 2014). Moreover, using Granger causality, Kullmann et al. (2014) showed decreased effective connectivity in AN within the cognitive control system notably in the bilateral inferior frontal gyrus, and increased connectivity within salience processing regions like the orbitofrontal gyri and insulae (Kullmann et al., 2014). Our results add to these resting state fMRI studies by showing altered patterns of connectivity during task performance in ill as well as recovered AN patients and thereby confirm the potential of functional connectivity measures for characterizing eating disorder patients.

### Caveats and limitations

There are some caveats and limitations to this study. We did not find amygdala or OFC activation to food images in AN as shown in other studies (Ellison et al., 1998; Uher et al., 2003, 2004; Killgore and Yurgelun-Todd, 2005; Goldstone et al., 2009; Siep et al., 2009). This may be due to the small size of the amygdala, combined with low power due to our small sample size, and heterogeneity in AN patients, as we included both restrictive and purging AN individuals.

Furthermore, Brooks et al.’s hypothesis describes a difference in brain activation between the restrictive and purging type, with an increase in BU activation and decrease in TD activation in the purging individuals. We included both restrictive and purging individuals, with the majority being restrictive (60% AN and 71% REC). This might have influenced the increased activation in the BU activation.

These differences may also be due to the use of a different paradigm; our participants were asked to imagine eating, tasting the foods, and using items on the images, instead of passively looking at them. This cognitive processing paradigm may account for a lack of TD control as patients did not need to actively inhibit any desire to eat. Furthermore, we were unable to verify cognitive engagement with the images. The increased visual cortex activation (in the food versus NF group contrasts), however, suggests engagement in the images during the experiment.

## Conclusion

In this study, we could not fully confirm the hypothesis of Brooks et al. (2012a), as we did not find food related deactivation of the BU processing in AN patients. We did confirm an increase in TD information processing. Activation in the VPA suggest stronger inhibition to food-directed attention, supporting a stronger TD control in AN.

This study shows that even though the REC group was long-term recovered for a mean duration of 4.7 years, they still show large differences in food information processing compared to HC. We found REC to have the most significant differences in food-cue activation in BU, TD, and VPA when compared with HC. AN showed fewer significant differences compared to HC than REC did.

This might be explained by diminished sensory processing in AN as a result of neglecting the presented food cues. The AN group is less reactive to food cues, knowing they will avoid consuming the shown foods. This might explain why we did find some activation in the BU areas, without a strong TD response. In REC, sensory processing might be somewhat recovered, showing a stronger TD response reflecting a stronger aversion to food cues, which they have successfully overcome.

## Conflict of Interest Statement

The authors declare that the research was conducted in the absence of any commercial or financial relationships that could be construed as a potential conflict of interest.

## Supplementary Material

The Supplementary Material for this article can be found online at http://www.frontiersin.org/Journal/10.3389/fnbeh.2015.00046/abstract

## Footnotes

^1^http://www.fil.ion.ucl.ac.uk/spm/software/spm8/

^2^http://marsbar.sourceforge.net/
