# Supplementary material for: Altered Food-Cue Processing in Chronically Ill and Recovered Women with Anorexia Nervosa
Source: Front Behav Neurosci. 2015 Feb 27;9:46. doi: 10.3389/fnbeh.2015.00046 (PMC4342866; doi:10.3389/fnbeh.2015.00046)
Supplement: Supplementary file 1 [file datasheet_1.zip › Appendix_1.docx]

| fROI | Peak voxel coordinates of fROI  X Y Z | | | Cluster size (voxels) F Peak voxel | |
| --- | --- | --- | --- | --- | --- |
| *Bottom-up* |  |  |  |  |  |
| *Striatum* |  |  |  |  |  |
| R caudate nucleus | 10 | 8 | 14 | 25 | 6.5 |
| Putamen | - |  |  |  |  |
| Accumbens | - |  |  |  |  |
| Pallidum | - |  |  |  |  |
| L hippocampus | -22 | -16 | -14 | 55 | 8.92 |
| R hippocampus | -18 | -4 | -14 | 55 | 12.95 |
| Amygdala | - |  |  |  |  |
| Hypothalamus | 6 | -4 | -10 | 11 | 4.28 |
| *Cerebellum* |  |  |  |  |  |
| Vermis | 6 | -36 | -6 | 9 | 8.07 |
| Vermis 2 | 6 | -76 | -14 |  | 15.27 |
| L cerebellum | -30 | -60 | -22 | 198 | 23.51 |
| R cerebellum | 30 | -68 | -22 | 180 | 23.14 |
| R cerebellum 2 | 6 | -52 | -38 | 9 | 8.76 |
| *Insula* |  |  |  |  |  |
| R. Insula | 38 | 8 | -14 | 18 | 6.96 |
| *Top-down* |  |  |  |  |  |
| *OFC* |  |  |  |  |  |
| L middle frontal gyrus | -34 | 4 | 50 | 220 | 8.64 |
| R middle frontal gyrus | 38 | 40 | 10 | 37 | 14.04 |
| R middle frontal gyrus 2 | 42 | 20 | 34 | 24 | 5.33 |
| Dorsolateral prefrontal cortex | - |  |  |  |  |
| M prefrontal cortex | - |  |  |  |  |
| Anterior cingulate cortex | - |  |  |  |  |
| *Visual processing* |  |  |  |  |  |
| *Visual cortex* |  |  |  |  |  |
| R cuneus | 18 | -80 | 30 | 20 | 6.17 |
| L precuneus | -10 | -52 | 54 | 52 | 12.32 |
| R precuneus | 14 | -52 | 38 | 14 | 3.79 |
| R superior frontal gyrus | 14 | 40 | 50 | 33 | 6.71 |
| R lingual gyrus | 18 | -92 | -10 | 300 | 54.77 |
| *Parietal cortex* |  |  |  |  |  |
| L inferior parietal cortex | -26 | -60 | 42 | 40 | 8.13 |
| R inferior parietal cortex | 34 | -52 | 42 | 66 | 15.20 |
| L post central gyrus | -42 | -28 | 42 |  |  |
| L superior temporal gyrus | -50 | -8 | -10 | 254 | 12.42 |
| R superior temporal gyrus | 54 | -8 | -2 | 199 | 12.36 |
| R posterior cingulate cortex | 14 | -56 | 14 | 66 | 11.34 |
