# Supplementary material for: Altered Food-Cue Processing in Chronically Ill and Recovered Women with Anorexia Nervosa
Source: Front Behav Neurosci. 2015 Feb 27;9:46. doi: 10.3389/fnbeh.2015.00046 (PMC4342866; doi:10.3389/fnbeh.2015.00046)
Supplement: Supplementary file 1 [file datasheet_1.zip › Appendix_2.docx]

**Appendix 2.**

**Functional analysis: Cluster H2**


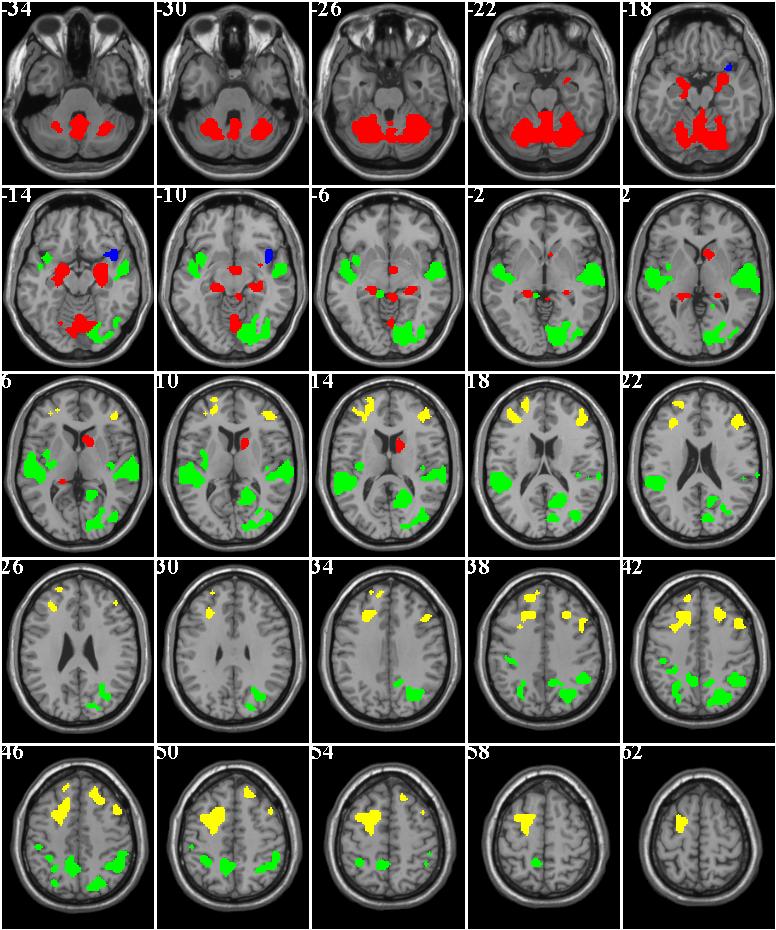


^red = Bottom-up; Yellow = Top-down; blue = insula; green = Visual processing areas^
