# Supplementary material for: Altered Food-Cue Processing in Chronically Ill and Recovered Women with Anorexia Nervosa
Source: Front Behav Neurosci. 2015 Feb 27;9:46. doi: 10.3389/fnbeh.2015.00046 (PMC4342866; doi:10.3389/fnbeh.2015.00046)
Supplement: Supplementary file 1 [file datasheet_1.zip › Appendix_3.docx]

**Appendix 3. Correlation Matrices**

1. **Healthy controls**

|  | R caudate nucleus | L hippocampus | R hippocampus | Hypothalamus | vermis | Vermis 2 | L cerebellum | R cerebellum | R cerebellum 2 | R insula | L middle frontal gyrus | R middle frontal gyrus | R middle frontal gyrus 2 | R cuneus | L precuneus | R precuneus | R sup frontal gyrus | R lingual gyrus | L inferior parietal cortex | R inf parietal cortex | L post central gyrus | L sup temporal gyrus | R sup temporal gyrus | R post cingulate cortex |
| --- | --- | --- | --- | --- | --- | --- | --- | --- | --- | --- | --- | --- | --- | --- | --- | --- | --- | --- | --- | --- | --- | --- | --- | --- |
| R caudate nucleus | 1 | .433 | .561 | .859 | .522 | .550 | .508 | .340 | -.161 | .439 | -.094 | .577 | .514 | .261 | .481 | .239 | .405 | .599 | .122 | .378 | -.004 | .519 | .550 | .529 |
| L hippo-campus | .433 | 1 | .835 | .515 | .811 | .721 | .731 | .821 | .451 | .704 | .629 | .629 | .468 | .620 | .512 | .127 | .659 | .750 | .664 | .530 | .357 | .590 | .360 | .755 |
| R hippo-campus | .561 | .835 | 1 | .561 | .745 | .714 | .842 | .749 | .305 | .829 | .127 | .127 | .655 | .766 | .591 | .430 | .752 | .800 | .499 | .693 | .140 | .750 | .541 | .869 |
| Hypothalamus | .859 | .515 | .561 | 1 | .540 | .533 | .534 | .428 | -.284 | .461 | .168 | .747 | .435 | .429 | .517 | .268 | .639 | .646 | .435 | .440 | .204 | .540 | .547 | .537 |
| vermis | .522 | .811 | .745 | .540 | 1 | .577 | .524 | .487 | .148 | .694 | .259 | .464 | .270 | .478 | .560 | .324 | .570 | .636 | .513 | .426 | .275 | .431 | .176 | .634 |
| Vermis 2 | .550 | .721 | .714 | .533 | .577 | 1 | .786 | .744 | .367 | .610 | .044 | .547 | .433 | .394 | .230 | -.086 | .600 | .792 | .471 | .417 | .284 | .698 | .509 | .455 |
| L cerebellum | .508 | .731 | .842 | .534 | .524 | .786 | 1 | .850 | .268 | .678 | .123 | .776 | .647 | .667 | .324 | .103 | .691 | .926 | .536 | .637 | .099 | .677 | .581 | .623 |
| R cerebellum | .340 | .821 | .749 | .428 | .487 | .744 | .850 | 1 | .396 | .557 | .336 | .605 | .503 | .632 | .256 | -.112 | .634 | .830 | .512 | .383 | .358 | .608 | .440 | .656 |
| R cerebellum 2 | -.161 | .451 | .305 | -.284 | .148 | .367 | .268 | .396 | 1 | .378 | .315 | -.049 | .174 | .161 | -.078 | -.214 | -.064 | .159 | .258 | .184 | .161 | .123 | .103 | .170 |
| R insula | .439 | .704 | .829 | .461 | .694 | .610 | .678 | .557 | .378 | 1 | .265 | .676 | .619 | .584 | .590 | .510 | .655 | .668 | .689 | .817 | .281 | .679 | .605 | .579 |
| L middle frontal gyrus | -.094 | .629 | .127 | .168 | .259 | .044 | .123 | .336 | .315 | .265 | 1 | .250 | .032 | .212 | .287 | -.149 | .116 | .245 | .607 | .115 | .384 | .106 | .190 | .133 |
| R middle frontal gyrus | .577 | .629 | .127 | .747 | .464 | .547 | .776 | .605 | -.049 | .676 | .250 | 1 | .762 | .656 | .584 | .390 | .712 | .781 | .686 | .833 | .167 | .711 | .705 | .600 |

|  | R caudate nucleus | L hippocampus | R hippocampus | Hypothalamus | vermis | Vermis 2 | L cerebellum | R cerebellum | R cerebellum 2 | R insula | L middle frontal gyrus | R middle frontal gyrus | R middle frontal gyrus 2 | R cuneus | L precuneus | R precuneus | R sup frontal gyrus | R lingual gyrus | L inferior parietal cortex | R inf parietal cortex | L post central gyrus | L sup temporal gyrus | R sup temporal gyrus | R post cingulate cortex |
| --- | --- | --- | --- | --- | --- | --- | --- | --- | --- | --- | --- | --- | --- | --- | --- | --- | --- | --- | --- | --- | --- | --- | --- | --- |
| R middle frontal gyrus 2 | .514 | .468 | .655 | .435 | .270 | .433 | .647 | .503 | .174 | .619 | .032 | .762 | 1 | .616 | .613 | .493 | .395 | .626 | .307 | .790 | -.089 | .684 | .727 | .639 |
| R cuneus | .261 | .620 | .766 | .429 | .478 | .394 | .667 | .632 | .161 | .584 | .212 | .656 | .616 | 1 | .628 | .498 | .621 | .698 | .527 | .636 | .343 | .629 | .529 | .796 |
| L precuneus | .481 | .512 | .591 | .517 | .560 | .230 | .324 | .256 | -.078 | .590 | .287 | .584 | .613 | .628 | 1 | .726 | .505 | .463 | .470 | .657 | .308 | .660 | .638 | .638 |
| R precuneus | .239 | .127 | .430 | .268 | .324 | -.086 | .103 | -.112 | -.214 | .510 | -.149 | .390 | .493 | .498 | .726 | 1 | .443 |  |  |  |  |  |  |  |
| R superior frontal gyrus | .405 | .659 | .752 | .639 | .570 | .600 | .691 | .634 | -.064 | .655 | .116 | .712 | .395 | .621 | .505 | .443 | 1 | .625 | .688 | .633 | .339 | .613 | .429 | .565 |
| R lingual gyrus | .599 | .750 | .800 | .646 | .636 | .792 | .926 | .830 | .159 | .668 | .245 | .781 | .626 | .698 | .463 | .086 | .625 | 1 | .555 | .558 | .276 | .734 | .647 | .642 |
| L inf parietal cortex | .122 | .664 | .499 | .435 | .513 | .471 | .536 | .512 | .258 | .689 | .607 | .686 | .307 | .527 | .470 | .254 | .688 | .555 | 1 | .677 | .564 | .463 | .453 | .273 |
| R inf parietal cortex | .378 | .530 | .693 | .440 | .426 | .417 | .637 | .383 | .184 | .817 | .115 | .833 | .790 | .636 | .657 | .679 | .633 | .558 | .677 | 1 | .104 | .657 | .673 | .524 |
| L post central gyrus | -.004 | .357 | .140 | .204 | .275 | .284 | .099 | .358 | .161 | .281 | .384 | .167 | -.089 | .343 | .308 | -.022 | .339 | .276 | .564 | .104 | 1 | .346 | .309 | .125 |
| L sup temporal gyrus | .519 | .590 | .750 | .540 | .431 | .698 | .677 | .608 | .123 | .679 | .106 | .711 | .684 | .629 | .660 | .329 | .613 | .734 | .463 | .657 | .346 | 1 | .860 | .660 |
| R sup temporal gyrus | .550 | .360 | .541 | .547 | .176 | .509 | .581 | .440 | .103 | .605 | .190 | .705 | .727 | .529 | .638 | .322 | .429 | .647 | .453 | .673 | .309 | .860 | 1 | .432 |
| R post cingulate cortex | .529 | .755 | .869 | .537 | .634 | .455 | .623 | .656 | .170 | .579 | .133 | .600 | .639 | .796 | .638 | .433 | .565 | .642 | .273 | .524 | .125 | .660 | .432 | 1 |

**Appendix 3. Correlation Matrices**

1. **Anorexia Nervosa**

|  | R caudate nucleus | L hippocampus | R hippocampus | Hypothalamus | vermis | Vermis 2 | L cerebellum | R cerebellum | R cerebellum 2 | R insula | L middle frontal gyrus | R middle frontal gyrus | R middle frontal gyrus 2 | R cuneus | L precuneus | R precuneus | R sup frontal gyrus | R lingual gyrus | L inferior parietal cortex | R inf parietal cortex | L post central gyrus | L sup temporal gyrus | R sup temporal gyrus | R post cingulate cortex |
| --- | --- | --- | --- | --- | --- | --- | --- | --- | --- | --- | --- | --- | --- | --- | --- | --- | --- | --- | --- | --- | --- | --- | --- | --- |
| R caudate nucleus | 1 | .741 | .824 | .687 | .744 | .593 | .516 | .680 | .419 | .760 | .653 | .401 | .619 | .580 | .018 | .435 | .464 | .646 | .156 | .463 | .472 | .826 | .763 | .412 |
| L hippo-campus | .741 | 1 | .827 | .734 | .846 | .777 | .751 | .810 | .557 | .641 | .601 | .472 | .671 | .593 | .149 | .636 | .613 | .757 | .529 | .689 | .571 | .736 | .671 | .736 |
| R hippo-campus | .824 | .827 | 1 | .719 | .937 | .784 | .716 | .892 | .621 | .861 | .688 | .306 | .372 | .401 | .277 | .579 | .613 | .648 | .313 | .538 | .458 | .661 | .617 | .643 |
| Hypothalamus | .687 | .734 | .719 | 1 | .710 | .547 | .565 | .712 | .483 | .527 | .392 | .283 | .439 | .651 | .357 | .569 | .447 | .556 | .294 | .350 | .580 | .579 | .649 | .644 |
| vermis | .744 | .846 | .937 | .710 | 1 | .770 | .730 | .783 | .531 | .804 | .642 | .199 | .373 | .480 | .347 | .661 | .696 | .623 | .366 | .549 | .355 | .612 | .571 | .735 |
| Vermis 2 | .593 | .777 | .784 | .547 | .770 | 1 | .908 | .781 | .787 | .695 | .630 | .168 | .399 | .550 | .495 | .736 | .728 | .761 | .725 | .603 | .428 | .683 | .664 | .851 |
| L cerebellum | .516 | .751 | .716 | .565 | .730 | .908 | 1 | .719 | .773 | .704 | .679 | .249 | .457 | .470 | .441 | .319 | .736 | .777 | .754 | .564 | .434 | .591 | .504 | .827 |
| R cerebellum | .680 | .810 | .892 | .712 | .783 | .781 | .719 | 1 | .634 | .699 | .662 | .368 | .388 | .329 | .190 | .574 | .505 | .670 | .431 | .487 | .503 | .570 | .516 | .649 |
| R cerebellum 2 | .419 | .557 | .621 | .483 | .531 | .787 | .773 | .634 | 1 | .555 | .599 | .243 | .404 | .269 | .405 | .485 | .464 | .616 | .684 | .644 | .522 | .426 | .449 | .549 |
| R insula | .760 | .641 | .861 | .527 | .804 | .695 | .704 | .699 | .555 | 1 | .865 | .248 | .336 | .425 | .227 | .548 | .728 | .510 | .311 | .331 | .289 | .693 | .598 | .574 |
| L middle frontal gyrus | .653 | .601 | .688 | .392 | .642 | .630 | .679 | .662 | .599 | .865 | 1 | .393 | .572 | .337 | .003 | .517 | .622 | .561 | .479 | .434 | .271 | .618 | .466 | .464 |
| R middle frontal gyrus | .401 | .472 | .306 | .283 | .199 | .168 | .249 | .368 | .243 | .248 | .393 | 1 | .692 | .201 | -.283 | .064 | .092 | .585 | .240 | .396 | .645 | .399 | .245 | .093 |
| R middle frontal gyrus 2 | .619 | .671 | .372 | .439 | .373 | .399 | .457 | .388 | .404 | .336 | .572 | .692 | 1 | .523 | -.144 | .370 | .303 | .709 | .489 | .700 | .474 | .662 | .555 | .317 |
| R cuneus | .580 | .593 | .401 | .651 | .480 | .550 | .470 | .329 | .269 | .425 | .337 | .201 | .523 | 1 | .416 | .731 | .621 | 504 | .440 | .249 | .371 | .806 | .876 | .709 |

|  | R caudate nucleus | L hippocampus | R hippocampus | Hypothalamus | vermis | Vermis 2 | L cerebellum | R cerebellum | R cerebellum 2 | R insula | L middle frontal gyrus | R middle frontal gyrus | R middle frontal gyrus 2 | R cuneus | L precuneus | R precuneus | R sup frontal gyrus | R lingual gyrus | L inferior parietal cortex | R inf parietal cortex | L post central gyrus | L sup temporal gyrus | R sup temporal gyrus | R post cingulate cortex |
| --- | --- | --- | --- | --- | --- | --- | --- | --- | --- | --- | --- | --- | --- | --- | --- | --- | --- | --- | --- | --- | --- | --- | --- | --- |
| L precuneus | .018 | .149 | .277 | .357 | .347 | .495 | .441 | .190 | .405 | .227 | .003 | -.283 | -.144 | .416 | 1 | .638 | .504 | .231 | .414 | .215 | -.037 | .221 | .411 | .688 |
| R precuneus | .435 | .636 | .579 | .569 | .661 | .736 | .319 | .574 | .485 | .548 | .517 | .064 | .370 | .731 | .638 | 1 | .843 | .436 | .540 | .410 | .065 | .704 | .738 | .887 |
| R superior frontal gyrus | .464 | .613 | .613 | .447 | .696 | .728 | .736 | .505 | .464 | .728 | .622 | .092 | .303 | .621 | .504 | .843 | 1 | .378 | .411 | .262 | .005 | .732 | .615 | .825 |
| R lingual gyrus | .646 | .757 | .648 | .556 | .623 | .761 | .777 | .670 | .616 | .510 | .561 | .585 | .709 | .504 | .231 | .436 | .378 | 1 | .727 | .730 | .693 | .595 | .554 | .615 |
| L inf parietal cortex | .156 | .529 | .313 | .294 | .366 | .725 | .754 | .431 | .684 | .311 | .479 | .240 | .489 | .440 | .414 | .540 | .411 | .727 | 1 | .633 | .457 | .339 | .388 | .692 |
| R inf parietal cortex | .463 | .689 | .538 | .350 | .549 | .603 | .564 | .487 | .644 | .331 | .434 | .396 | .700 | .249 | .215 | .410 | .262 | .730 | .633 | 1 | .410 | .423 | .443 | .439 |
| L post central gyrus | .472 | .571 | .458 | .580 | .355 | .428 | .434 | .503 | .522 | .289 | .271 | .645 | .474 | .371 | -.037 | .065 | .005 | .693 | .457 | .410 | 1 | .329 | .379 | .281 |
| L sup temporal gyrus | .826 | .736 | .661 | .579 | .612 | .683 | .591 | .570 | .426 | .693 | .618 | .399 | .662 | .806 | .221 | .704 | .732 | .595 | .339 | .423 | .329 | 1 | .924 | .632 |
| R sup temporal gyrus | .763 | .671 | .617 | .649 | .571 | .664 | .504 | .516 | .449 | .598 | .466 | .245 | .555 | .876 | .411 | .738 | .615 | .554 | .388 | .443 | .379 | .924 | 1 | .659 |
| R post cingulate cortex | .412 | .736 | .643 | .644 | .735 | .851 | .827 | .649 | .549 | .574 | .464 | .093 | .317 | .709 | .688 | .887 | .825 | .615 | .692 | .439 | .281 | .632 | .659 | 1 |

**Appendix 3. Correlation Matrices**

1. **Recovered**

|  | R caudate nucleus | L hippocampus | R hippocampus | Hypothalamus | vermis | Vermis 2 | L cerebellum | R cerebellum | R cerebellum 2 | R insula | L middle frontal gyrus | R middle frontal gyrus | R middle frontal gyrus 2 | R cuneus | L precuneus | R precuneus | R sup frontal gyrus | R lingual gyrus | L inferior parietal cortex | R inf parietal cortex | L post central gyrus | L sup temporal gyrus | R sup temporal gyrus | R post cingulate cortex |
| --- | --- | --- | --- | --- | --- | --- | --- | --- | --- | --- | --- | --- | --- | --- | --- | --- | --- | --- | --- | --- | --- | --- | --- | --- |
| R caudate nucleus | 1 | .105 | .333 | .423 | .191 | .483 | .437 | .356 | .193 | .389 | .299 | .487 | .226 | .333 | .049 | -.258 | .354 | .567 | .251 | .226 | .163 | -.091 | -.314 | .180 |
| L hippo-campus | .105 | 1 | .772 | .587 | .758 | .080 | .314 | .424 | -.073 | .192 | .231 | .159 | .009 | -.423 | -.144 | -.258 | -.276 | .633 | .710 | .526 | .448 | .466 | .234 | -.228 |
| R hippo-campus | .333 | .772 | 1 | .425 | .771 | .350 | .532 | .601 | -.029 | .433 | .226 | .402 | .329 | -.090 | -.180 | -.333 | -.001 | .861 | .864 | .697 | .501 | .562 | .363 | -.079 |
| Hypothalamus | .423 | .587 | .425 | 1 | .374 | .354 | .542 | .628 | .265 | .253 | .640 | .668 | .174 | -.283 | -.191 | -.331 | -.087 | .595 | .533 | .332 | .486 | .167 | -.216 | -.379 |
| vermis | .191 | .758 | .771 | .374 | 1 | .295 | .373 | .446 | .063 | .275 | -.051 | .185 | .266 | -.208 | .011 | -.041 | -.228 | .579 | .560 | .656 | .088 | .297 | .274 | -.009 |
| Vermis 2 | .483 | .080 | .350 | .354 | .295 | 1 | .882 | .661 | .651 | .509 | .017 | .604 | .795 | -.158 | -.025 | -.031 | .214 | .428 | .200 | .510 | .032 | -.239 | -.365 | .255 |
| L cerebellum | .437 | .314 | .532 | .542 | .373 | .882 | 1 | .894 | .503 | .424 | .346 | .795 | .737 | -.329 | -.250 | -.283 | .029 | .636 | .384 | .520 | .374 | .077 | -.269 | -.047 |
| R cerebellum | .356 | .424 | .601 | .628 | .446 | .661 | .894 | 1 | .244 | .381 | .553 | .801 | .600 | -.319 | -.225 | -.286 | -.085 | .664 | .451 | .540 | .399 | .375 | -.027 | -.149 |
| R cerebellum 2 | .193 | -.073 | -.029 | .265 | .063 | .651 | .503 | .244 | 1 | .347 | -.200 | .366 | .780 | -.357 | -.042 | .178 | -.041 | .118 | -.070 | .144 | -.238 | -.560 | -.616 | .038 |
| R insula | .389 | .192 | .433 | .253 | .275 | .509 | .424 | .381 | .347 | 1 | .089 | .250 | .493 | .108 | .302 | .264 | .196 | .409 | .278 | .661 | -.061 | .145 | .116 | .452 |
| L middle frontal gyrus | .299 | .231 | .226 | .640 | -.051 | .017 | .346 | .553 | -.200 | .089 | 1 | .546 | -.099 | .094 | -.098 | -.287 | .193 | .393 | .255 | .182 | .580 | .496 | .080 | -.282 |
| R middle frontal gyrus | .487 | .159 | .402 | .668 | .185 | .604 | .795 | .801 | .366 | .250 | .546 | 1 | .532 | -.204 | -.474 | -.507 | .004 | .683 | .393 | .223 | .520 | .060 | -.390 | -.444 |
| R middle frontal gyrus 2 | .226 | .009 | .329 | .174 | .266 | .795 | .737 | .600 | .780 | .493 | -.099 | .532 | 1 | -.337 | .000 | .140 | .013 | .322 | .169 | .471 | -.124 | -.120 | -.248 | .169 |
| R cuneus | .333 | -.423 | -.090 | -.283 | -.208 | -.158 | -.329 | -.319 | -.357 | .108 | .094 | -.204 | -.337 | 1 | .241 | .092 | .615 | -.028 | -.135 | -.078 | -.151 | -.018 | .266 | .356 |
| L precuneus | .049 | -.144 | -.180 | -.191 | .011 | -.025 | -.250 | -.225 | -.042 | .302 | -.098 | -.474 | .000 | .241 | 1 | .872 | .439 | -.468 | -.291 | 451 | -.590 | .076 | .328 | .848 |

|  | R caudate nucleus | L hippocampus | R hippocampus | Hypothalamus | vermis | Vermis 2 | L cerebellum | R cerebellum | R cerebellum 2 | R insula | L middle frontal gyrus | R middle frontal gyrus | R middle frontal gyrus 2 | R cuneus | L precuneus | R precuneus | R sup frontal gyrus | R lingual gyrus | L inferior parietal cortex | R inf parietal cortex | L post central gyrus | L sup temporal gyrus | R sup temporal gyrus | R post cingulate cortex |
| --- | --- | --- | --- | --- | --- | --- | --- | --- | --- | --- | --- | --- | --- | --- | --- | --- | --- | --- | --- | --- | --- | --- | --- | --- |
| R precuneus | -.258 | -.258 | -.333 | -.331 | -.041 | -.031 | -.283 | -.286 | .178 | .264 | -.287 | -.507 | .140 | .092 | .872 | 1 | .329 | -.577 | -.527 | .341 | -.812 | -.172 | .150 | -.735 |
| R superior frontal gyrus | .354 | -.276 | -.001 | -.087 | -.228 | .214 | .029 | -.085 | -.041 | .196 | .193 | .004 | .013 | .615 | .439 | .329 | 1 | .009 | -.127 | .315 | -.120 | -.139 | -.032 | .507 |
| R lingual gyrus | .567 | .633 | .861 | .595 | .579 | .428 | .636 | .664 | .118 | .409 | .393 | .683 | .322 | -.028 | -.468 | -.577 | .009 | 1 | .750 | .432 | .603 | .300 | -.037 | -.330 |
| L inf parietal cortex | .251 | .710 | .864 | .533 | .560 | .200 | .384 | .451 | -.070 | .278 | .255 | .393 | .169 | -.135 | -.291 | -.527 | -.127 | .750 | 1 | .457 | .689 | .584 | .368 | -.277 |
| R inf parietal cortex | .226 | .526 | .697 | .332 | .656 | .510 | .520 | .540 | .144 | .661 | .182 | .223 | .471 | -.078 | 451 | .341 | .315 | .432 | .457 | 1 | .045 | .409 | .358 | .452 |
| L post central gyrus | .163 | .448 | .501 | .486 | .088 | .032 | .374 | .399 | -.238 | -.061 | .580 | .520 | -.124 | -.151 | -.590 | -.812 | -.120 | .603 | .689 | .045 | 1 | .501 | .094 | -.582 |
| L sup temporal gyrus | -.091 | .466 | .562 | .167 | .297 | -.239 | .077 | .375 | -.560 | .145 | .496 | .060 | -.120 | -.018 | .076 | -.172 | -.139 | .300 | .584 | .409 | .501 | 1 | .811 | -.039 |
| R sup temporal gyrus | -.314 | .234 | .363 | -.216 | .274 | -.365 | -.269 | -.027 | -.616 | .116 | .080 | -.390 | -.248 | .266 | .328 | .150 | -.032 | -.037 | .368 | .358 | .094 | .811 | 1 | .266 |
| R post cingulate cortex | .180 | -.228 | -.079 | -.379 | -.009 | .255 | -.047 | -.149 | .038 | .452 | -.282 | -.444 | .169 | .356 | .848 | -.735 | .507 | -.330 | -.277 | .452 | -.582 | -.039 | .266 | 1 |
